# One case of left atrial arterial intimal sarcoma

**DOI:** 10.1016/j.ijscr.2025.112091

**Published:** 2025-11-03

**Authors:** Zhou Qiao, Zhang Mengsi

**Affiliations:** Department of Pathology, Wuhan Asia General Hospital, Wuhan, 430000, China

**Keywords:** Case report, Soft tissue sarcoma, Endometrial sarcoma, Angiosarcoma

## Abstract

**Background:**

To explore the clinical and pathological characteristics as well as immunophenotypes of arterial endometrial sarcoma, and to improve the understanding of this disease. Due to the poor efficacy of late stage radiotherapy and chemotherapy, early diagnosis and surgical eradication are of great significance.

**Method:**

Clinical data of a case of arterial intimal sarcoma occurring in the left atrium were summarized, HE and immunohistochemical staining were performed to observe its morphological and immunophenotypic characteristics, and relevant literature was reviewed.

**Result:**

The patient was a middle-aged female. Cardiac CT showed a local right bulging of the left atrial septum, while cardiac ultrasound showed an enlargement of the left atrium. Several moderately uneven echogenic tumor reflections were observed in the atrial cavity, with a large amplitude of oscillation. The local echo of the interventricular septum is thin and soft, forming a tumor like structure that swells towards the right atrium. Under the microscope, the tumor cells appear spindle shaped overall, with obvious cellular atypia. Nuclear division and sparse and dense areas of cells can be seen, with some areas showing mucinous stroma and others showing abundant cells growing around blood vessels. Immunohistochemistry confirmed MDM2 positivity.

**Conclusion:**

Endometrial sarcoma is a rare malignant tumor that occurs in the endometrium and may originate from multifunctional stem cells beneath the endometrium. Endometrial sarcoma IS is often a high-grade undifferentiated sarcoma that can differentiate in multiple directions. Combining specific disease sites with morphological diagnosis is not difficult.

## 1 Introduction

Secondary cardiac tumors are much more common compared with primary cardiac tumors (100–1000 times). Majority of the primary cardiac tumors are benign (∼75 %), and nearly 50 % of these are atrial myxomas [1]. Twenty-five percent of primary cardiac tumors are malignant, and 95 % of these are due to sarcoma, the remaining 5 % are lymphomas [2]. Angiosarcoma is the most common type and accounts for around 40 % of sarcomas, others include undifferentiated sarcoma (24 %), malignant fibrous histiocytoma (14–24 %), leiomyosarcoma (8 %), osteosarcoma (6 %), and least of all, spindle cell sarcoma [3].

Arterial intimal sarcoma (AIS) is a malignant mesenchymal tumor originating from the aorta and pulmonary artery, first reported by Mandelstaml in 1923 during autopsy [4]. This tumor grows in the vascular lumen, blocks the vascular lumen, and can form a tumor thrombus and spread to surrounding organs. It can also occur in the heart and is the most common soft tissue sarcoma of the heart [5].

In this report, we describe a case of an extremely rare malignant primary cardiac tumor—Endometrial sarcoma of left atrial artery. Cardiac intimal sarcomas are remarkably aggressive and least reported type of primary malignant tumors of the heart, and their clinical presentation is variable, ranging from common complains such as fatigue and dyspnoea to syncope. This article aims to improve the diagnosis of IS by analyzing clinical data, morphology, immunohistochemistry, and reviewing relevant literature.

## 2 Data and methods

### 2.1 General information collection

In May 2024, a difficult case was diagnosed with left atrial intimal sarcoma.

### 2.2 Method

Collect imaging and surgical data of cases; The submitted specimen is a surgical specimen, which is fixed in 10 % neutral formalin, dehydrated, embedded in paraffin, sliced, stained with HE, and observed for pathological morphology after surgery.

CD31, S100, Facter VIII, Ki-67 The first antibody was purchased from Zhongshan Company, and the specific operation steps were carried out according to the instructions of the reagent kit. All the work has been reported in line with the SCARE criteria.

## 3 Case presentation/results

### 3.1 Clinical imaging and surgical data

The patient is a 35 years old female with no medical history. She came to our heart center for treatment due to “palpitations for a week”. In the past week, there have been intermittent and unexplained palpitations, which can be relieved for several minutes each time. Sometimes accompanied by dizziness. Two days ago, I experienced pain in my right lower limb without any cause. A cardiac computed tomography (CT) scan was performed in our hospital, which showed local rightward bulging of the left atrial septum (as shown in Fig. 1A). Cardiac ultrasound revealed a space occupying lesion in the left atrium, suggesting the possibility of myxoma, with a large amplitude of tumor movement (as shown in Fig. 1B). The outpatient department admitted patients with “severe mitral regurgitation with relative stenosis”+“mild tricuspid regurgitation”+“atrial septal dilation tumor”. During the operation, no adhesions were observed in the pericardium, and the left heart was enlarged. The anterior and posterior mitral valves were extensively adhered to mucus tissue, and the valve annulus was enlarged with stenosis and incomplete closure. Diffuse tumor tissue was attached to the left atrial sidewall, left atrial appendage, right lower pulmonary vein, and left upper and lower pulmonary veins (as shown in Fig. 1C).

**Fig. 1A:**
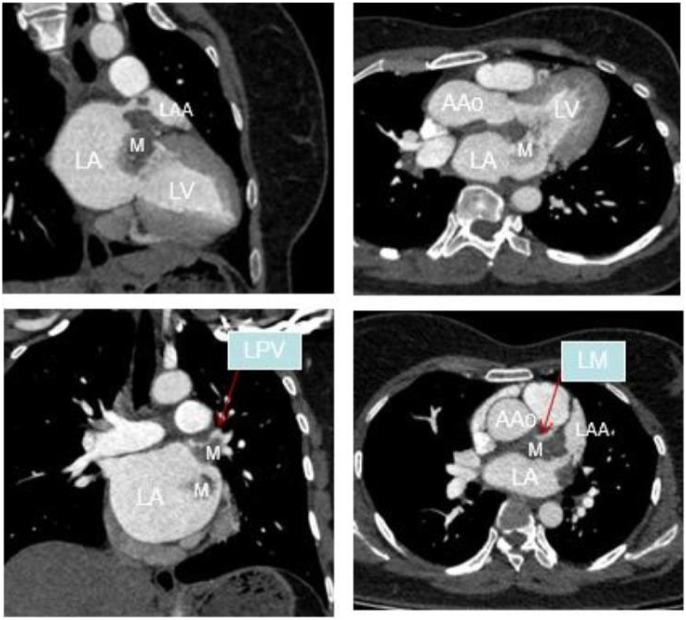
Computed tomography of arterial intimal sarcoma.

**Fig. 1B:**
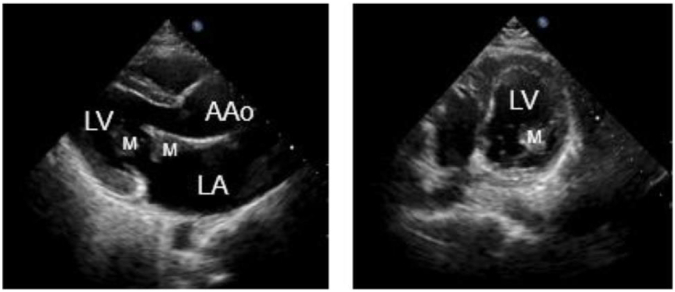
Ultrasound examination of arterial endometrial sarcoma.

**Fig. 1C:**
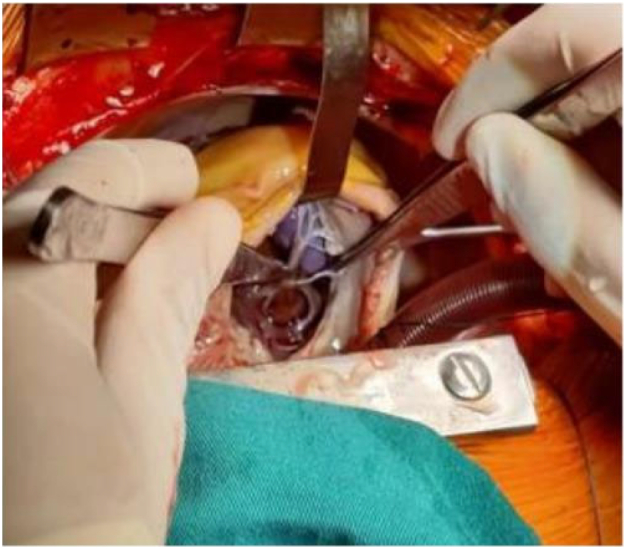
Surgical resection of arterial endometrial sarcoma during surgery.

### 3.2 Visually observe

**Fig. 2A:**
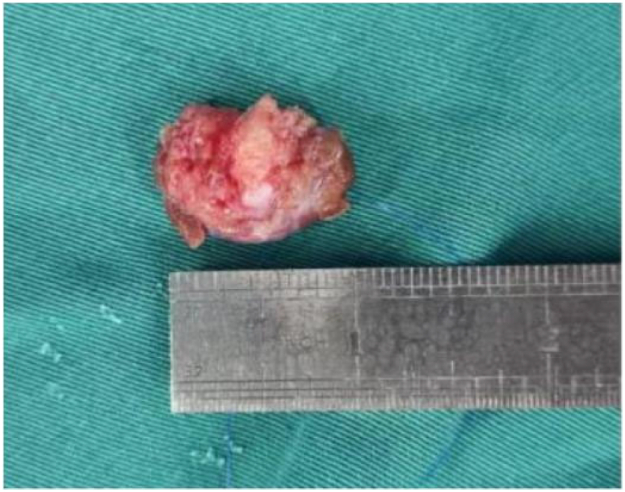
General appearance of arterial intimal sarcoma.

A pile of gray white gray yellow dark brown fragmented tissue, with a total size of 6.0 cm × 5.5 cm × 3.5 cm. Some sections are gray white and slightly hard, while others are gray yellow and jelly like (as shown in Fig. 2A).

### 3.3 Under the microscope

Tumor cells appear spindle shaped overall, with obvious cellular atypia, visible nuclear division, and sparse and dense areas of cells; Mucous like stroma can be seen in some areas, and cells are abundant in some areas, with more cells growing around blood vessels (as shown in Fig. 3A, Fig. 3B, Fig. 3C).

**Fig. 3A:**
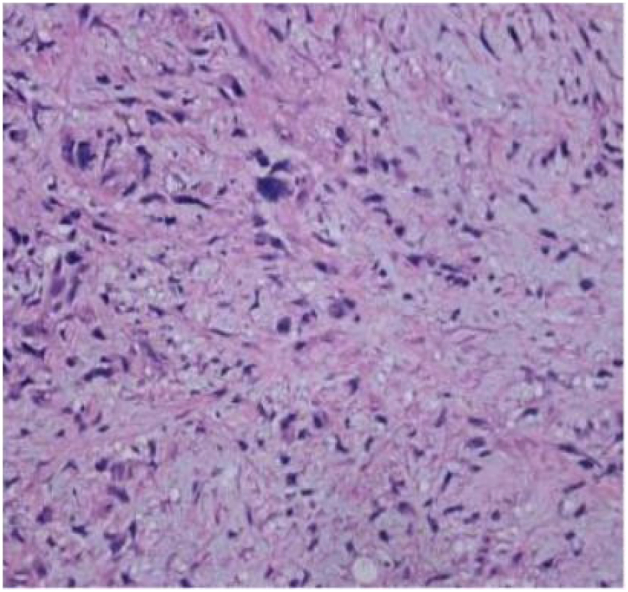
The overall shape of tumor cells is spindle shaped.

**Fig. 3B:**
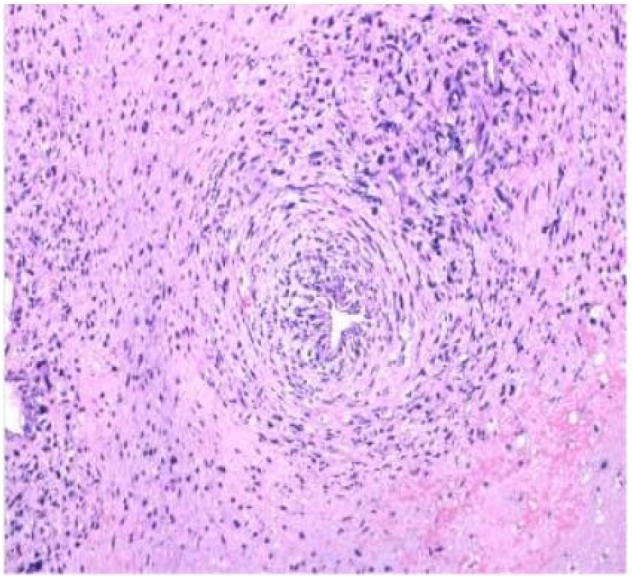
Obvious cellular atypia, visible nuclear division, visible sparse and dense areas of cells.

**Fig. 3C:**
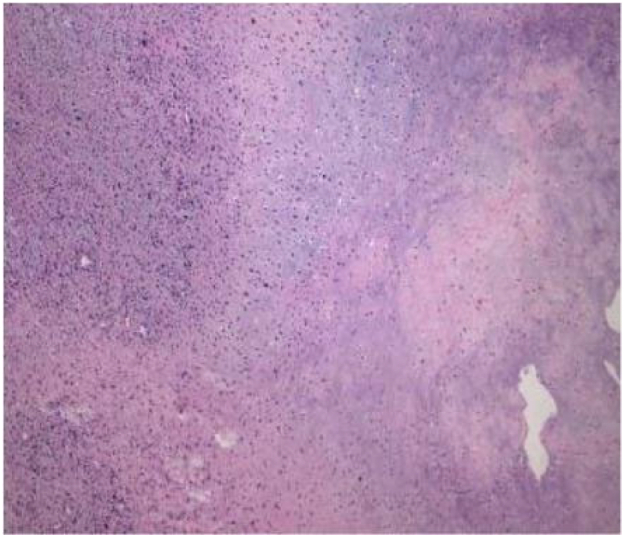
More cells can be seen growing around blood vessels.

### 3.4 Immunophenotypes

P16 (+), MDM2 (+), CDK4 (+), Desmin (partially+), Caldesmon (partially+), SMA (+), CD34 (−), ERG (partially+), MyoD1 (−), Myogenin (−), CD31 (−), S100 (−), Facter VIII (−), Ki-67 (Li:20 %), as shown in Fig. 4A, Fig. 4B, Fig. 4C.

**Fig. 4A:**
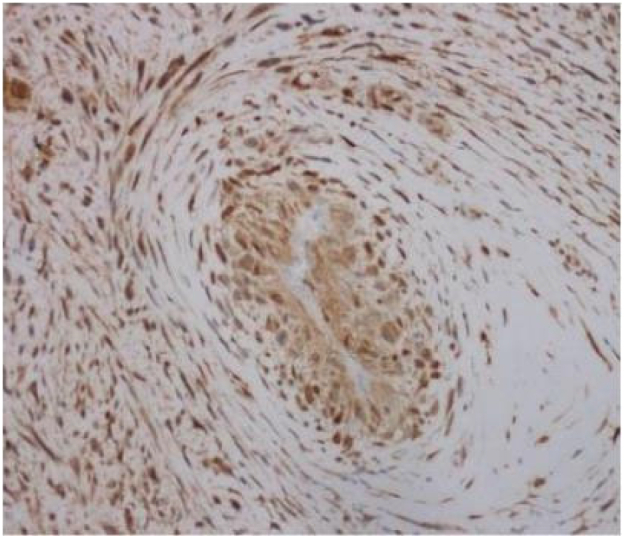
CDK4.

**Fig. 4B:**
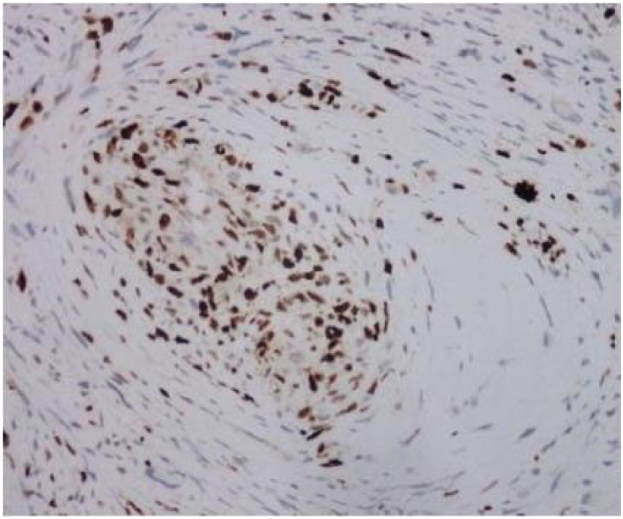
Ki67.

**Fig. 4C:**
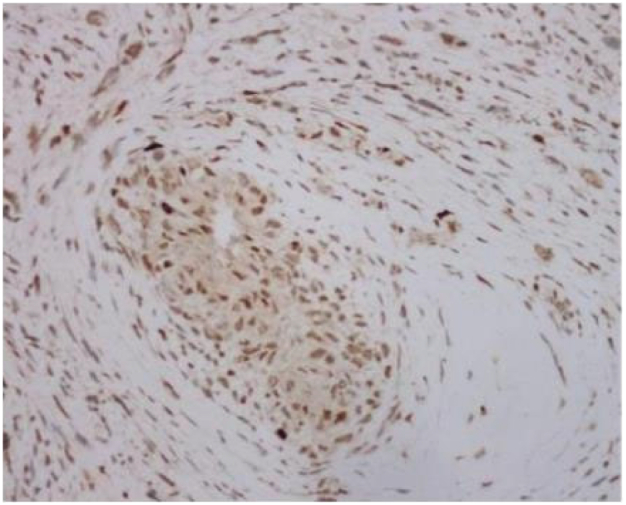
MDM2.

### 3.5 Pathological diagnosis (cardiac mass)

Malignant mesenchymal tumor, with a tendency towards arterial intimal sarcoma.

## 4 Discussion

Cardiac sarcoma can present with sudden, intermittent, or positional symptoms, including chest discomfort, difficulty breathing, sitting breathing, coughing or fainting, heart failure, pericardial effusion and tamponade, arrhythmia, valve abnormalities or blockages, depending on the involved atrium or ventricle. Non-invasive imaging methods such as transthoracic or transesophageal echocardiography are commonly used for diagnosis. CT and MRI can be used to fully evaluate tumors occupying the entire cardiac cavity, tumors infiltrating the myocardium or pericardium, or tumors involving adjacent large blood vessels [6]. Patients often experience a rapid deterioration in health, and the prognosis is very poor with a mean survival of 3 months to 1 year. Cardiac intimal sarcomas are encountered more commonly in the large arterial blood vessels, including the pulmonary artery and aorta, and are extremely rare in the heart. Due to the rarity of arterial intimal sarcoma occurring in the heart and the lack of specificity in clinical manifestations, we attempted to unify our understanding of this disease through pathological testing.

Burke et al [7] referred to those originating from the wall of the vessel as wall sarcoma and those originating from the endometrium as endometrial sarcoma. This case is a typical arterial endometrial sarcoma originating from the endometrium. In recent years, AIS related immunohistochemistry has shown that tumors can express different differentiation markers, suggesting that they are highly likely to originate from multifunctional stem cells under the intima of blood vessels [8], with the potential for multi-directional differentiation.IS is attached to the vascular wall in a polypoid shape. Most histologically, it is difficult to classify and presents as an undifferentiated sarcoma, composed of spindle shaped cells or obese cells with obvious atypia and active nuclear division. Cytoplasmic vacuoles can be seen, and the morphology is similar to undifferentiated sarcoma derived from myofibroblasts, sometimes containing epithelioid cells. Immunohistochemistry is non-specific, usually Vmentin diffuse positive and SMA focal positive. When the tumor has clear heterologous differentiation, corresponding immune markers can be expressed. Comparing genomic hybridization, it was found that the common 12q13–14 region was obtained and amplified, which showed MDM2 gene amplification, leading to immunohistochemical overexpression of MDM2 [5].

Under the microscope in this case, it can be seen that the tumor cells are spindle shaped as a whole, with obvious cellular atypia, visible nuclear division, and sparse and dense areas of cells; Mucous like stroma can be seen in some areas, and cells are abundant in some areas, with more cells growing around blood vessels. The tumor grows diffusely and cannot be ruled out as originating from blood vessels, fat, smooth muscle, and striated muscle. Further immunohistochemistry was performed to assist in diagnosis, and the results showed: P16 (+), MDM2 (+), CDK4 (+), Desmin (partially+), Caldesmon (partially+), SMA (+), CD34 (−), ERG (partially+), MyoD1(−), Myogenin(−), CD31(−), S100(−), Facter VIII(−), Ki-67(Li:20 %).Malignant tumor can be clearly identified, MDM2 positivity is clear, considering arterial endometrial sarcoma, but whether MDM2 is amplified requires further molecular support, and according to the article “Revised Surgical CAse REport (SCARE) guideline: An update for the age of Artificial Intelligence” [9]，the final diagnosis is malignant mesenchymal tumor (cardiac mass), leaning towards arterial endometrial sarcoma.

## Ethical approval

The study was performed at The Department of Pathology, Wuhan Asia General Hospital, Wuhan, China. Wuhan Asia General Hospital Ethical Committee approved the study (Approval number: WAGHMECLW-2024004).

## Guarantor

Guarantor is ZhouQiao.

## Funding

No funding was received.

## Author contribution

ZHouQiao: Conception and design, the drafting of the paper, revising it critically for intellectual content.

ZhangMengsi: Analysis and interpretation of the data.

## Conflict of interest statement

The authors declare no conflicts of interest.
